# The Importance of Patient-Centered Care in Colon Cancer Patients With Severe Mental Illness

**DOI:** 10.7759/cureus.16386

**Published:** 2021-07-14

**Authors:** Angel Guan, Vincent Ma, Arjun C Bakshi, Davin Agustines

**Affiliations:** 1 College of Osteopathic Medicine of the Pacific, Western University of Health Sciences, Pomona, USA; 2 College of Medicine, California Northstate University, Elk Grove, USA; 3 Psychiatry Department, Olive View University of California Los Angeles Medical Center, Los Angeles, USA

**Keywords:** colon cancer, schizophrenia, communication, whipple procedure, failed colonoscopy

## Abstract

There is an abundance of literature that highlights the importance of patient-centered communication with cancer patients requiring surgical intervention. While the need for communication for patients requiring surgery is well understood, less attention is brought to patients with severe mental illnesses. More literature is needed to highlight the importance and application of patient-centered care for patients suffering from both severe mental illness and cancer requiring surgical intervention. It is unclear if poor communication between patients and cancer-care specialists is part of the reason for the underlying discrepancy. Efforts to reduce this discrepancy may be worth considering as a priority for health care systems. We present a case of a 63-year-old man with schizophrenia who received a late cancer diagnosis after a missed screening, resulting in an extensive surgical resection for colon cancer. We explore the possibility of careful communication between the treating physician, patient, and patient’s caretakers potentially preventing the delay in his cancer diagnosis. Effective communication is especially important with mental health patients because of its effect on long-term physical and mental outcomes. We hope to further the discussion on how to better cater to this specific population of patients undergoing cancer surgery.

## Introduction

Colon cancer is the third most diagnosed cancer in the United States and the second most common leading cause of cancer death [1]. Schizophrenia is a severe mental illness that affects approximately one percent of the world population and is characterized by behaviors including but not limited to delusions, hallucinations, disorganized thinking and speech, apathy, anhedonia, and catatonia [2]. Previous studies have concluded that compared to the non-mentally ill population, patients with schizophrenia receive less cancer screenings, have unknown cancer staging at the time of diagnosis, receive no chemotherapy, and have worse post-surgical outcomes [3-5]. The underlying reason behind these conclusions is multifactorial. It is likely a combination of the ramifications of severe mental health issues on a patient’s ability to follow medical recommendations, systemic discrimination in health care against patients with mental illness, and challenges navigating financial and bureaucratic hurdles in the health care system [6]. Many patients with mental illness are treated at facilities that are separate from general hospital services, increasing the difficulty of communication between psychiatric care teams and medical-surgical care teams [5]. Patients with mental illness often exhibit deficits in reality testing, resulting in a refusal to accept diagnostic care, as well as decreased trust in the health care system in general. This can make the consent process very difficult to attain. We present a case of a man living with schizophrenia who was later diagnosed with stage III colon cancer and required surgical resection. Verbal and written consent for this case report was obtained from both the patient and the patient’s conservator.

## Case presentation

Mr. X is a 63-year-old male with a 40-year history of schizophrenia who was well controlled on clozapine for his psychosis and disorganized thought. Due to the severity of his mental illness, his sister is his conservator, which means that she makes all of his legal and medical decisions. Mr. X has a family history of colon cancer, where his mother was diagnosed with colon cancer in her 60s. The course of his disease started with symptoms of recurrent diarrhea and right lower quadrant abdominal pain. Since Mr. X is conserved, Mr. X’s sister gave informed consent for Mr. X to undergo an initial colonoscopy. Mr. X did not understand the instructions on how to perform the bowel prep and he did not finish drinking the bowel prep solution. This resulted in a failed colonoscopy due to poor visualization as there was too much stool in his colon. A secondary test utilizing barium enema revealed an apple core stenosing mass distal to the cecum. The discovery of an apple core lesion of the colon can be due to several etiologies, including but are not limited to inflammatory bowel disease, infection, and cancer [7]. A second colonoscopy was performed but did not find the lesion that was described in the barium enema and did not identify any additional lesions. Mr. X was symptomatically treated for pain and diarrhea and his symptoms spontaneously abated. There was no further evaluation until 10 months later when Mr. X experienced a resurgence of abdominal pain. Computed tomography of the abdomen and pelvis revealed a 17.6-cm mass originating from the cecum and extending superiorly, invading both the duodenum and pancreatic head. He was diagnosed with stage III colon cancer and the surgeon informed both the patient and his caregivers that he required a complicated surgery as a potential cure for his cancer.

After undergoing an open Whipple procedure with right colectomy en bloc resection, he was hospitalized for eight days. The psychiatric consultation-liaison team worked closely with the surgical team to help Mr. X maintain psychiatric stability. In the months after discharge, Mr. X suffered physical complications from his surgery. These complications included severe malnutrition that led to total parenteral nutrition and gastronomy tube placement. Mr. X then suffered from paralytic ileus, followed by severe bacterial pneumonia. As a result, Mr. X had to be admitted and discharged repeatedly from multiple hospitals and skilled nursing facilities (SNF). During one of his stays at an SNF, he refused to take his clozapine; over time, he became agitated, aggressive, and began physically assaulting the nursing home staff and his family caretakers.

Mr. X then demonstrated both disorganized speech and thought, ultimately declaring suicidal ideation. At that point, Mr. X was admitted to our inpatient psychiatric ward for suicidal ideation and decompensation of his chronic schizophrenia. Mr. X’s sister reported that he was on clozapine prior to admission and that he was uncooperative because of the weekly blood draws. Thus, he was started on oral olanzapine, negating the need for weekly blood draws, as part of a regimen targeted toward reducing his psychosis and impulsiveness. However, Mr. X refused to take oral olanzapine. Repeated attempts to restart oral antipsychotic medications failed as Mr. X refused to take oral medications alone; only after both intramuscular and oral formulations of olanzapine were utilized did Mr. X regain compliance with psychiatric treatment. After several weeks within the inpatient psychiatric unit, Mr. X’s schizophrenia symptoms lessened to the point where he no longer expressed aggression and suicidal ideation.

## Discussion

Mr. X had a very strong family support system, underwent an operation at a well-renowned tertiary care center, and was followed by both general surgery and psychiatry teams during his hospitalization. Despite these beneficial factors, his lack of understanding of how to do proper bowel preparation resulted in delayed diagnosis and multiple medical and psychiatric complications. A cancer diagnosis radically changes a patient’s life. Our patient did not grasp the weight of this diagnosis due to his severe mental illness and thus, was heavily reliant on his care teams to clearly communicate the importance and process of screenings. In addition, patients with cancer are often required to go through an extensive screening process to prepare for the surgical resection that will hopefully provide a cure. Not only is this a difficult process for people without mental illness, but it can also be especially difficult for people with schizophrenia.

Since Mr. X was conserved by his sister, she was heavily involved with the consent process and possessed the power of attorney for Mr. X. She made the decision to move forward with the various diagnostic screenings and surgical intervention. Although Mr. X was present during these medical visits, he did not understand the preparation process for his colonoscopy and did not fully evacuate his bowels for visualization. Ultimately, this resulted in a delayed diagnosis and an extensive surgical resection of a large tumor with several postoperative complications. Undergoing surgery is both physically and emotionally stressful, and it requires immense patience and motivation to achieve a smooth recovery. The post-surgical period requires the patient to ambulate early, limit the use of pain medications as much as possible, and use incentive spirometry to prevent respiratory complications. This requires a great deal of cooperation from the patient, which many patients with severe mental illness cannot achieve without extensive communication and behavioral incentives. As important as it is to build rapport and listen to each patient, it is especially important to gain the trust of a patient with schizophrenia because of their low threshold for misinterpretation of information and their sensitivity to stress, as seen in our patient [8]. In addition, many patients with schizophrenia display symptoms of negative affect, which can render a patient unable to take the appropriate steps for medical treatment. Patients living with schizophrenia often have disorganized thinking and speech, which decreases their ability to communicate effectively and thus, are highly dependent on their physician’s ability to communicate and explain the diagnostic and treatment process each step of the way [9]. Patients with mental illness require extra time and support while undergoing treatment for their colon cancer. Thus, having care teams cognizant of the intricacies involved in building rapport with severely mentally ill patients may cause drastic changes in surgical outcomes and help to prevent adverse events.

## Conclusions

This case illustrates the importance of patient-centered care for patients suffering from both severe mental illness and cancer requiring surgical intervention. Patients with schizophrenia live with positive and negative symptoms that make it difficult for them to follow medical recommendations, thus resulting in later diagnoses and unfortunately, more extensive recoveries from surgery. To mitigate these complications, physicians should utilize a multidisciplinary approach including the psychiatrist, oncologists, and surgeons to work together in helping patients such as Mr. X recover from cancer treatment. Taking time to get to know each patient and understand the motivations behind their behavior may prove to be essential in providing quality care to prevent adverse outcomes and ultimately, improve patients’ quality of life.
